# Supplementary material for: Foldclass and Merizo-search: scalable structural similarity search for single- and multi-domain proteins using geometric learning
Source: Bioinformatics. 2025 May 6;41(5):btaf277. doi: 10.1093/bioinformatics/btaf277 (PMC12122203; doi:10.1093/bioinformatics/btaf277)
Supplement: btaf277_Supplementary_Data [file btaf277_supplementary_data.zip › Merizo-search Supplementary Information.pdf]

Supplementary Information

Foldclass and Merizo-search: Scalable structural similarity search for single- and multi-domain proteins using geometric learning

Kandathil, S. M.[1], Lau, A. M.[1+], Buchan, D. W. A.[1] , and Jones, D. T.[1,2*]

Test of generalisation on unseen domains

We collected 7,427 novel fold domains identified in the Encyclopedia of Domains (TED; Lau et al., 2024), which were identified after multiple rounds of Foldseek-based structural clustering. Of these, 1,930 domains are singletons, so we used the remaining 5,497 domains as queries for Foldclass searches against the TED100 database (324 million domains) with *k* set to 21. Because these query domains are contained in the TED100 set, we exclude the top hit in each hit list (as these will always be a trivial match to the query), and consider the remaining *N* hits, where *N* = min(20, *cluster_size*), and *cluster_size* is the size of the cluster for which the query is the representative.

A total of 60304 hits were identified for the 5,497 queries. Of these, 33,946 hits for 4,333 queries had a TM-align score of 0.5 or greater, meaning that 78.8% of the domains could be matched to at least one true-positive hit. This indicates that Foldclass embeddings are reasonably consistent even in unseen regions of fold space, and that confirmatory TM-align runs are essential to validate the initial hits identified by the initial embedding-based search.

Analysis of single-domain hits unique to Merizo-search

We assessed single-domain hits that were unique to Merizo-search, and found that in a great number of cases, these additional hits were CATH superfamily relatives that are diverged in sequence relative to the query. Although Foldseek is also able to identify hits with low sequence identity to the query, the hits unique to Merizo-search often also contain significant indels relative to the query.

An example is shown below (Figure S1) for query CATH domain 155cA00, for which Merizo-search identifies 3dmiA00 and 3vrdA01 as hits, which Foldseek (in exhaustive TM-align mode) does not find. These hit domains are in the same CATH superfamily as the query (cytochrome *c*; 1.10.760.10) and can be aligned with TM-align score > 0.5 to the query. However, these hits have large deletions relative to the query, and this almost certainly affects Foldseek's ability to identify them. Even when Foldseek's "exhaustive" mode is selected (disabling the similar k-mer prefiltering step), it uses gapless alignment (on sequence and optionally 3Di strings) to identify an initial set of putative hits. This step would be prone to missing low-sequence identity hits with significant indels relative to the query.

Since the final hit list is a subset of this initial set, the subsequent, more sensitive gapped alignment and other steps will not be able to recover the missing hits.

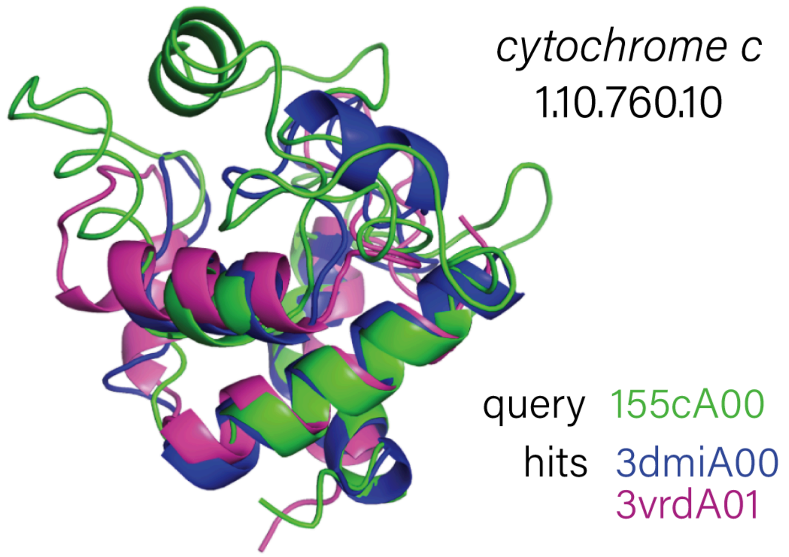

Figure S1. Examples of hits unique to Merizo-search. The query domain (155cA00 in green) and two hits (3dmiA00 in blue, and 3vrdA01 in magenta) are superposed using TM-align. These hits are found by Merizo-search, but not Foldseek, and both hits are in the same CATH superfamily as the query (cytochrome c; 1.10.760.10) and can be aligned with TM-align score > 0.5 to the query.
